# A single-cell interactome of human tooth germ from growing third molar elucidates signaling networks regulating dental development

**DOI:** 10.1186/s13578-021-00691-5

**Published:** 2021-10-02

**Authors:** Yueqi Shi, Yejia Yu, Yuqiong Zhou, Jun Zhao, Wenjie Zhang, Duohong Zou, Weichen Song, Shaoyi Wang

**Affiliations:** 1 Department of Oral Surgery, Shanghai Key Laboratory of Stomatology, National Center for Stomatology, National Clinical Research Center for Oral Diseases, College of Stomatology, Shanghai Ninth People’s Hospital, Shanghai Jiao Tong University School of Medicine, Shanghai Jiao Tong University, Shanghai, China; 2 Department of Stomatology, Tongren Hospital, Shanghai Jiao Tong University School of Medicine, Shanghai, 200336 China; 3 Department of Orthodontics, Shanghai Key Laboratory of Stomatology, National Center for Stomatology, National Clinical Research Center for Oral Diseases, College of Stomatology, Shanghai Ninth People’s Hospital, Shanghai Jiao Tong University School of Medicine, Shanghai Jiao Tong University, Shanghai, China; 4 Department of Prosthodontics, Shanghai Key Laboratory of Stomatology, National Center for Stomatology, National Clinical Research Center for Oral Diseases, College of Stomatology, Shanghai Ninth People’s Hospital, Shanghai Jiao Tong University School of Medicine, Shanghai Jiao Tong University, Shanghai, China; 5 Shanghai Mental Health Center, Shanghai Jiao Tong University School of Medicine, Shanghai, China

**Keywords:** Tooth germ, Single-cell sequencing, Signaling pathway, Osteoblast, BMP

## Abstract

**Background:**

Development of dental tissue is regulated by extensive cell crosstalk based on various signaling molecules, such as bone morphogenetic protein (BMP) and fibroblast growth factor (FGF) pathways. However, an intact network of the intercellular regulation is still lacking.

**Result:**

To gain an unbiased and comprehensive view of this dental cell interactome, we applied single-cell RNA-seq on immature human tooth germ of the growing third molar, discovered refined cell subtypes, and applied multiple network analysis to identify the central signaling pathways. We found that immune cells made up over 80% of all tooth germ cells, which exhibited profound regulation on dental cells via Transforming growth factor-β, Tumor necrosis factor (TNF) and Interleukin-1. During osteoblast differentiation, expression of genes related to extracellular matrix and mineralization was continuously elevated by signals from BMP and FGF family. As for the self-renewal of apical papilla stem cell, *BMP-FGFR1-MSX1* pathway directly regulated the G0-to-S cell cycle transition. We also confirmed that Colony Stimulating Factor 1 secreted from pericyte and TNF Superfamily Member 11 secreted from osteoblast regulated a large proportion of genes related to osteoclast transformation from macrophage and monocyte.

**Conclusions:**

We constructed the intercellular signaling networks that regulated the essential developmental process of human tooth, which served as a foundation for future dental regeneration engineering and the understanding of oral pathology.

**Supplementary Information:**

The online version contains supplementary material available at 10.1186/s13578-021-00691-5.

## Background

The human tooth and periodontal tissue emerge from the neural crest-derived ectomesenchyme of frontonasal, maxillary, and mandibular protrusions [1]. Tooth development is a long-term and complex biological process involving cell–cell and epithelial–mesenchymal interaction, cell differentiation, morphogenesis, tissue mineralization, and tooth eruption [2]. At the initial stage, cell proliferation activates in specific areas of the dental lamina. The proliferative epithelium then extends to the deep connective tissue, and the terminal cells proliferate and further develop into the enamel organ. At the same time, the ectomesenchyme cells under the proliferative epithelium also proliferate rapidly and gather around the epithelium. These locally proliferated epithelia and mesenchyme together form the tooth germ [3]. The tooth germ consists of three parts: (1) enamel organ originated from oral ectoderm and forms enamel; (2) dental papilla originated from ectomesenchyme, forms pulp and dentin; (3) dental follicle originated from ectomesenchyme, forms cementum, periodontal ligament and alveolar bone [4]. Undoubtedly, a comprehensive understanding of tooth development requires dissection of these tooth germ substructures.

Like all developmental processes, tooth development is regulated by a series of complex gene cascades, which drive the cells to enter a predetermined location and differentiate in a specific direction [2]. The formation of dental arch depends on the coordinated regulation of a variety of signaling molecules and location signals, which jointly regulate the development process of cell division rate, trend and direction of cell migration, cell differentiation and apoptosis. In the above process, a series of genes and signaling pathways play an important role in regulation, including Sonic hedgehog (Shh) pathway [5], Wingless-related integration (Wnt) pathway [6], fibroblast growth factors (FGF) pathway [7], Transforming growth factor-beta (TGF-β) [8] pathway and bone morphogenesis proteins (BMPs) family [9–11]. These signaling molecules bind to corresponding receptors and regulate the expression of specific genes. Specific functions elicited by activation of these pathways are noted during distinct phases of dental tissue differentiation, some of which are beneficial for cell stemness and proliferation (FGF, Shh) while others such as Wnt, TGF-β, and BMPs act in postnatal differentiation phases and promote polarization, migration, and calcification [1, 2].

Understanding the intact signaling network in tooth development is essential to dental regeneration engineering and clinical dentistry. So far, many functional studies have elucidated the components and processes of specific pathways [1, 2, 11–15], and the application of these insights has promoted translational medicine and the understanding of oral disease. The rapid advancement of single-cell RNA sequencing (scRNA-seq) and corresponding data analysis algorithms has provided a chance to draw this entire cell interactome. Scientists have applied scRNA-seq to draw the cell atlas of mouth dental development at various stages [16–18] and human periodontal tissue [19]. However, the comprehensive cell interactome of human tooth is still lacking.

**Fig. 1:**
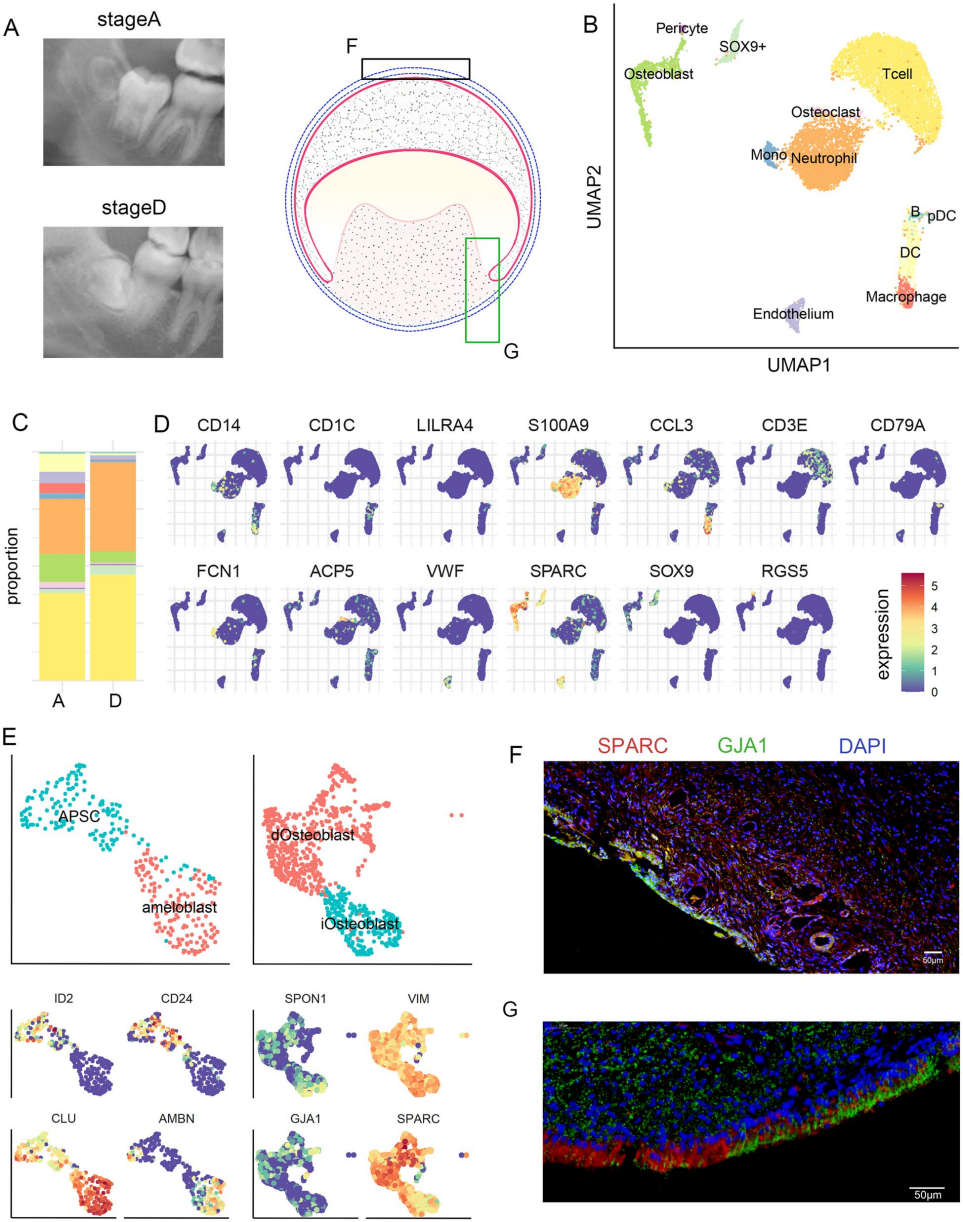
Cell type landscape of human tooth germ. **A** Oral panorama of two tooth germ samples at stage A and D used for scRNA-sEq. Schematics indicated the detail structure of tooth germ. Boxes indicated location of immunofluorescence (**F**, **G**). **B** UMAP projection of cell clustering results. **C** Proportion of each cell cluster in stage A and D tooth germ. **D** Expression patterns of selected cell markers. **E** Refined subtypes and their markers for SOX9^+^ cells and osteoblast. **F**, **G** Immunofluorescence result for SPARC and GJA1 expression in tooth germ

To achieve this goal, we applied scRNA-seq to tooth germs isolated from developing third molar of healthy volunteers who planned to have orthodontic treatment (Fig. 1A). In human, the third molar has the latest development time scale of all teeth, and properly retains immature tooth germ structure before one’s twenties, making it an ideal object for dental development research. In this study, we first identified refined dental cell subtypes in human tooth germ, discovered essential genes involved in dental cell differentiation and transformation, then constructed ligand-receptor-transcription factor networks that regulate these essential genes.

## Result

### Single-cell composition of human tooth germ

We isolated tooth germ tissue from two patients with different developmental statuses of left mandibular third molars (Fig. 1A and Additional file 1: Figure S1). One patient’s left mandibular third molar was at developmental stage A (calcification of cusp tips without coalescence of other calcifications). The other patient was at stage D (complete crown formation up to cementoenamel junction). The developmental status of the third molars was assessed using eight-stage developmental scoring (from A to H) proposed by Demirjian et al. [20]. Accordingly, cells from stage A expressed higher value of immature osteoblast markers like *SCUBE3*, whereas stage D cells expressed mature osteoblast markers like *SPARC* (Additional file 2: Table S1). We applied BD-seq on the dissected tooth germ and obtained transcriptome data for 9855 cells, which on average contained about 28,000 mapped reads per cell, after RNA quantification and quality control. Using Louvain method embedded in Seurat 3.0 R package [21], we partitioned all cells into 11 clusters (Fig. 1B). Various immune cells, including T cell (*CD3E*^+^), neutrophil (*S100A9*+), macrophage (*CCL3*^+^), monocytes (*FCN1*^+^) and dendritic cell (*CD1C*^+^) (Fig. 1B–D and Additional file 2: Table S1), consisted of nearly 83% of all cells. This large proportion of immune cell supported the notion that molar eruption involves immune activation of tooth sac which absorb the bone tissue covering the immature molar which obstruct the eruption. In the remaining cells, we identified *SPARC*^+^*RUNX2*^+^ osteoblast [22], *ACP5*^+^ osteoclast [23], *RGS5*^+^ pericytes and *VWF*^+^ endothelium. Lastly, we identified a population of *SOX9*^+^ cells that exhibited heterogeneous transcriptome characteristics (Additional file 2: Table S1).

To resolve this heterogeneity, we carried out further clustering analysis on the SOX9^+^ cells. As shown in Fig. 1E, *SOX9*^+^ cells consisted of two subpopulations: one expressed apical papilla stem cell (APSC) marker *CD24 *[24], and another expressed ameloblast marker *AMBN* and epithelium-associated gene *CLU *[25]. We therefore separated *SOX9*^+^ cells into APSC and ameloblast. Similarly, osteoblast (Fig. 1F) also consisted of two subpopulations, namely, immature and differentiated osteoblast (iOsteoblast and dOsteoblast, respectively). iOsteoblast expressed higher level of *VIM* that inhibited osteoblast differentiation [26], whereas dOsteoblast highly expressed *SPARC* that took part in osteogenesis [22] and *GJA1* that took part in osteoblast differentiation [27].

Since *SPARC* and *GJA1* expression is not limited to osteoblast in dental tissue, we further applied immunofluorescence to elucidate their spatial and cellular distribution in tooth germ. On the tip of dental sac (Fig. 1F and Additional file 1: Figure S2), which is proximal to the bone interface, *SPARC* and *GJA1* showed co-localization in the osteoblast. On the outer surface of dental papilla (Fig. 1G and Additional file 1: Figure S2), *SPARC* expressed in the odontoblast at the root end while *GJA1* expressed in the odontoblast at the crown end, with little co-localization at the middle. This result supported our classification of *SPARC*^+^*GJA1*^+^ cells as the mature osteoblast.

### T cell subpopulations and their intercellular interaction patterns

**Fig. 2:**
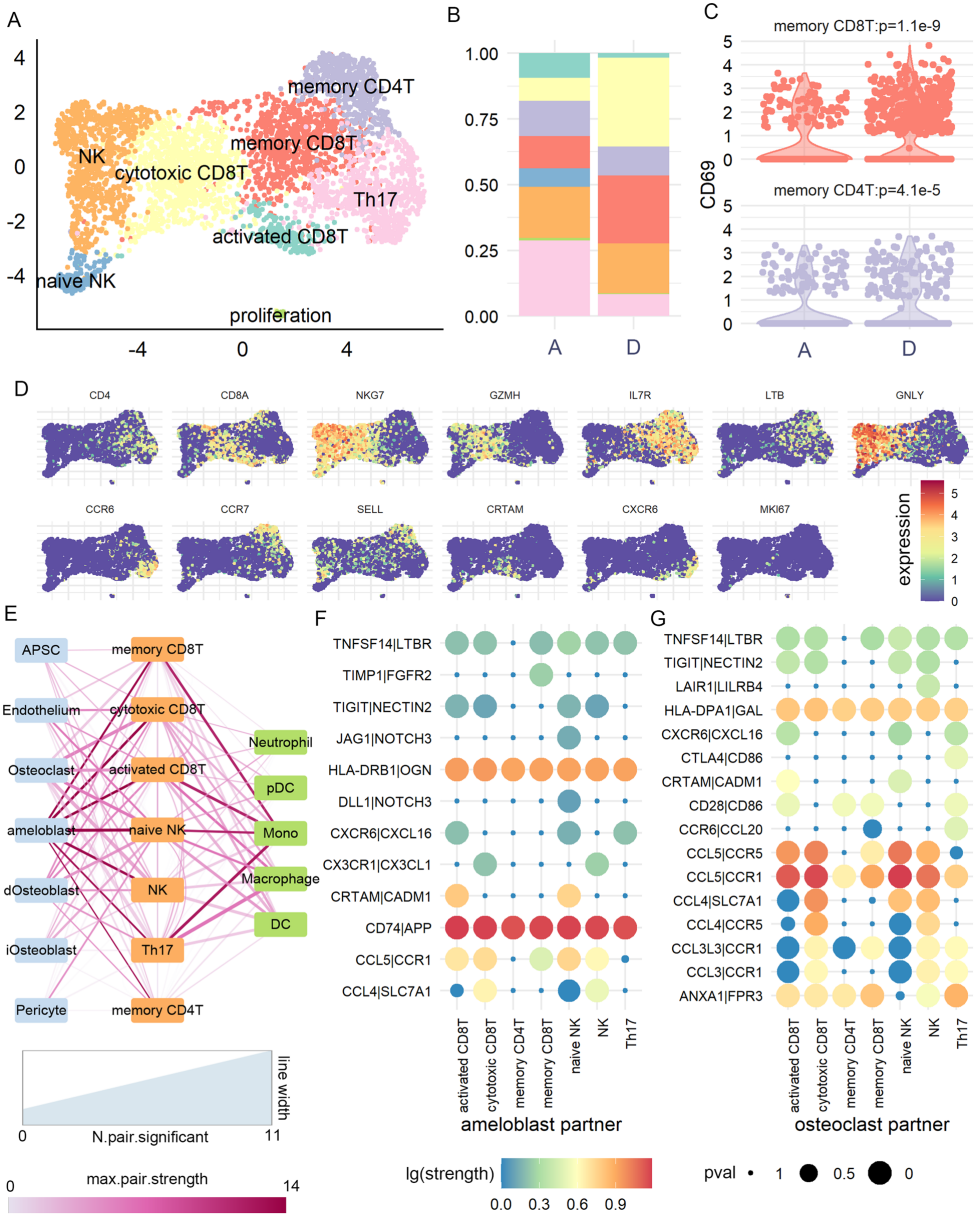
T cell subtypes and their cellular interaction patterns. **A** UMAP projection of T cell clustering results. **B** Proportion of each T cell subtype in stage A and D tooth germ. **C** Comparison of CD69 expression, a marker of tissue resident T cell, between two samples. **D** Expression patterns of selected cell markers. **E** Cellular interaction strength between each T subtype and all other cell types. Width of line indicated the number of ligand-receptor pairs reaching p < 0.05 in permutation test. Color of line indicated the maximal interaction strength between the two cell types. **F** Interaction strength of all ligand-receptor pairs reaching p > 0.05 in at least one cell type pairs. We only showed ligands secreted from T cell and corresponding receptors on another cell type (ameloblast and osteoclast)

Our single-cell data revealed that more than 42% of tooth germ cells were T cell (Fig. 1C), which presumably contained diverse subpopulations with important roles in tooth structure [28]. To analyze the role of these subpopulations, we applied Seurat cluster analysis on T cells and obtained eight sub-clusters (Fig. 2A–D). We first defined the cytotoxic T cells by *NKG7* and *GNLY* expression, and separated them into natural killer (NK) T and CD8T according to *CD8A* expression (Fig. 2D). We then defined memory T and Th17 by *IL7R*, *CCR6* and *CCR7* expression [29, 30]. Finally, we found small subpopulation of *CRTAM*^+^ activated CD8T, *SELL*^+^ naïve NK, and *MKI67*^+^ proliferation T cell. Separated by developmental period, we found that stage A tooth germ contained more Th17 (74% of Th17 came from stage A tooth germ) but less cytotoxic CD8T (17%, Fig. 2B). The proportion of tooth-residence memory cells was also higher in stage D tooth germ, reflected by higher expression of residence T cell marker CD69 [31] (permutation p < 4.1 × 10^− 5^, Fig. 2C).

We then applied CellPhoneDB [32] analysis to explore the subpopulation-specific intercellular signal transduction from T cell to other tooth germ cells. By summarizing the ligand-receptor pairs reaching significant threshold (“Method”), all T cell subpopulations showed strongest association with ameloblast (number of significant pairs = 2 to 9, association strength > 12; Fig. 2E), especially by *CD74-*to*-APP* and *HLADRB1-*to*-OGN* signaling pathways (Fig. 2F). Naïve NK and activated CD8T showed the most significant communication with ameloblast, and they exhibited subpopulation-specific pathways like *CXCR6*-to-*CXCL16* and *CRTAM*-to-*CADM1*. T cell also exhibited strong communication with osteoclast (number of significant pairs = 4 to 11, association strength = 2.5 to 6.1; Fig. 2E), especially by signals from *CCL3/CCL4/CCL5* to *CCR1/CCR5* (Fig. 2G). Interestingly, we observed *CTLA4*-to-*CD86* signaling which was unique to Th17 cell, where *CD86* was known to suppress osteoclast differentiation [33]. For other immune cells, T cell subpopulations showed divergent association with monocyte (number of significant pairs = 2 to 8, association strength = 1.6 to 12.3; Fig. 2E). These results suggested that human tooth germ contained diverse T cell subpopulation with distinct cell interaction patterns.

### Non-T immune cell subpopulations and their intercellular interaction patterns

**Fig. 3:**
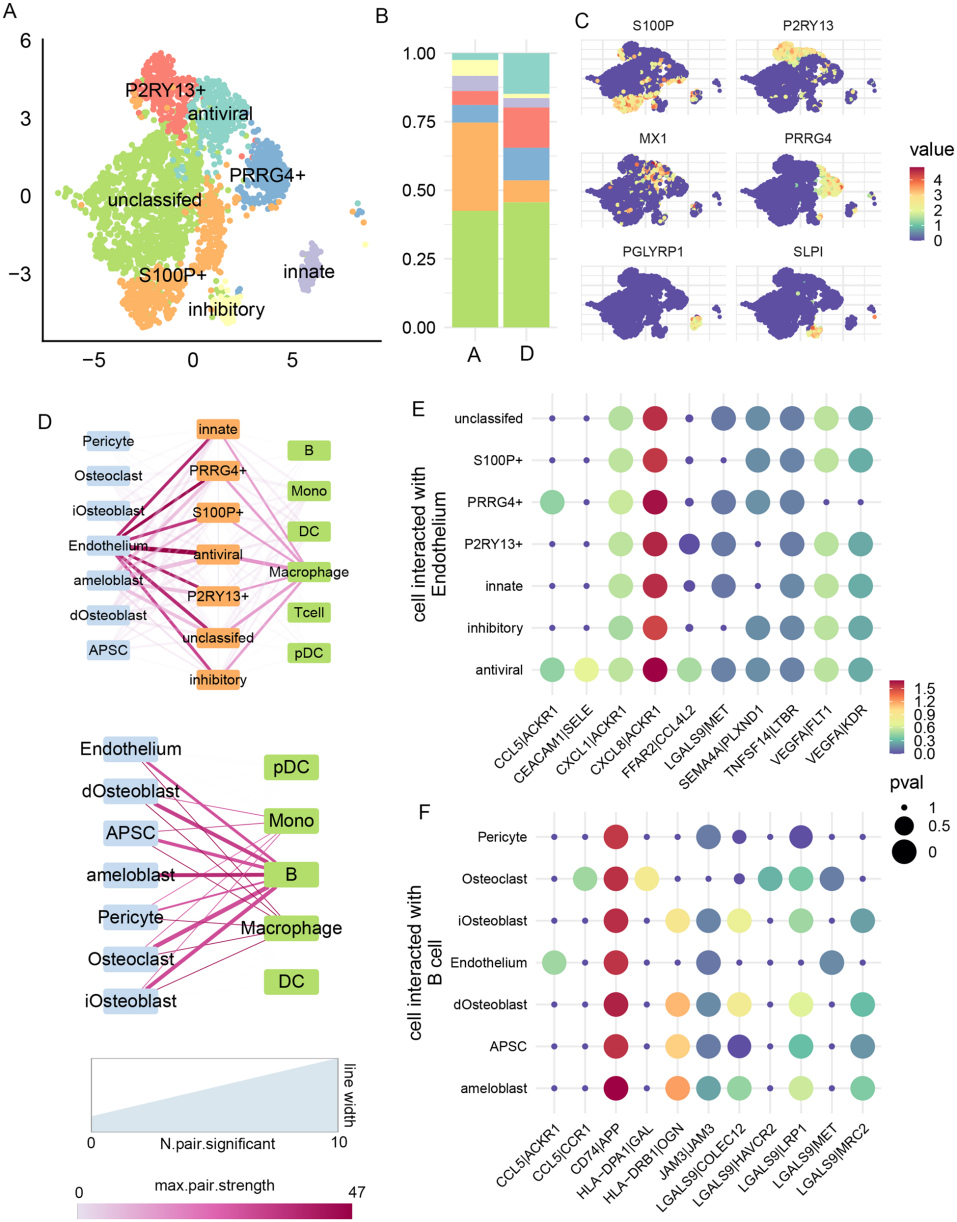
Non-T immune cell subtypes and their cellular interaction patterns. **A** UMAP projection of neutrophil clustering results. **B** Proportion of each neutrophil subtype. **C** Expression patterns of selected cell markers. **D** Cellular interaction strength between each neutrophil (up), other non-T immune cell (down), and all other cell types, similar to Fig. 2E. **E** Interaction strength of all ligand-receptor pairs reaching p > 0.05 in at least one cell type pairs. We only showed ligands secreted from neutrophil and corresponding receptors on endothelium. **F** Similar to **E**, but for interaction between B cell and other dental cells

Despite T cell subpopulations, other immune cells also play an important role in tooth development [34]. Since the proportion of neutrophil was profoundly larger than remaining immune cells (Fig. 1C), we first applied clustering analysis to dissect neutrophil subpopulation. As shown in Fig. 3A–C, we obtained eight subpopulations of neutrophil. They were classified by unique expression of genes related to neutrophil functions (Fig. 3C). We first identified *PGLYRP1*^+^ neutrophil with specific roles in innate immunity [35], as well as *MX1*^+^ antiviral neutrophil [36], and *SLPI*^+^ inhibitory neutrophil [37]. Another three subpopulations were labeled by *P2RY13*, *PRRG4* and *S100P*, all of which took part in neutrophil functions. Finally, a subcluster without specific markers but only expressed *S100A9* was annotated as “unclassified”. Separated by developmental period, stage A tooth germ contained more *S100P*^+^ neutrophils (71%) and less *P2RY13*^+^ (17%) and antiviral (9%) neutrophils.

We then applied CellPhoneDB [32] to analyze the signal transduction from non-T immune cells to dental cells. For neutrophil subpopulation (Fig. 3D, E), they were overwhelmingly associated with endothelium (number of significant pairs = 6 to 10, association strength > 30; strength between neutrophil and other cells < 18), especially via *ACKR1* which guided neutrophil migration [38]. Antiviral neutrophil showed the strongest association with endothelium (number of significant pairs = 10) and showed subpopulation-specific signaling of *CEACAM1*-to-*SELE*, which mediated neutrophil activation and migration [38]. For other immune cells, we found that B cells showed the strongest signaling transduction to dental cells (number of significant pairs = 2 to 7, association strength > 25), especially the *CD74*-to-*APP* pathway.

### Intercellular signaling network regulated osteoblast maturation

Having resolved all subpopulation for tooth germ cell types, we now had the opportunity to analyze the functional implication of intercellular signaling network. We started by delineating the process of osteoblast maturation, then analyzed whether this process was regulated by signals from other cells by using ligand-target [39] and transcription factor regulation network [40].

**Fig. 4:**
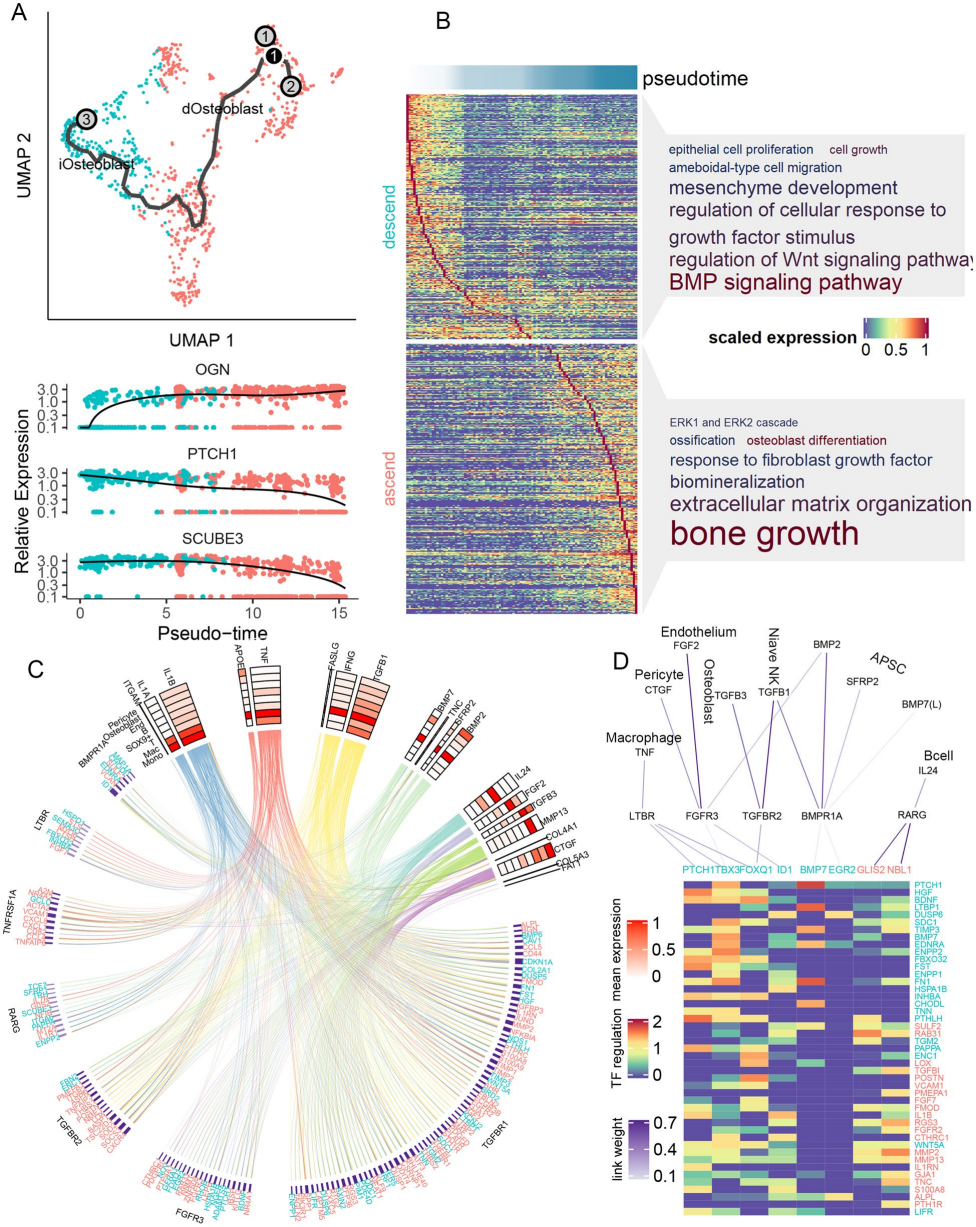
Intercellular signaling network regulating osteoblast differentiation. **A** Pseudotime analysis of osteoblast. Lower panel showed the expression trajectory along pseudotime for three genes as example. **B** GO-BP analysis of gene significantly altered along pseudotime. Font size showed the fold of enrichment of the pathway, and color showed the log p value of enrichment. **C** Circus plot of the signaling network. Upper part showed the ligands and their average expression in each cell types (red-scale heatmap). Lower part showed the target genes, and a line linking one ligand and one target indicated the regulation potentiality between them (predicted by NicheNet). Color of lines corresponded to the cell type with the highest ligand expression (color scales same as Fig. 1B). Targets are grouped according to their predicted upstream receptor (receptor names showed at the outer layer). Purple color bar indicated the predicted ligand-receptor association weight. **D** Transcription factors (TF) involved in osteoblast differentiation. From top to down: ligands from each cell types; receptors on the surface of osteoblast; transcription factor within osteoblast; regulation potentiality of TF on target genes

As shown in Fig. 4A, we applied monocle3 [41] pseudotime analysis on the osteoblast and observed a clear immature-to-differentiated lineage. We found that the expression of *ALPL*, *BGN* and *OGN*, genes related to mature osteoblast function [42], increased rapidly at the beginning of this lineage (Fig. 4A and Additional file 1: Figure S3). Other genes related to extracellular matrix formation such as *MMP2* and *COL12A1* also showed gradual increment throughout the lineage (Additional file 1: Figure S3). On the other hand, genes regulating the proliferation of osteoblast, such as *SCUBE1* and *PTCH1* (Fig. 4A), gradually decreased during this lineage. Taken all genes showing significant differential expression along pseudotime, we found a clear temporal cascade (Fig. 4B). By Gene Ontology (GO) analysis, we found that genes showing decreasing expression in the cascade mainly took part in Wnt signaling pathway (adjusted p value of GO analysis [GO P] = 4.41 × 10^− 11^, odds ratio [OR] = 5.21), mesenchymal development (GO P = 1.91 × 10^− 8^, OR = 5.08), BMP signaling pathway (GO P = 1.16 × 10^− 7^, OR = 6.12, Fig. 4B and Additional file 2: Table S2). Inversely, ascending genes took part in extracellular matrix organization (GO P = 5.24 × 10^− 31^, OR = 8.46), bone growth (GO P = 6.65 × 10^− 8^, OR = 13.49) and biomineralization (GO P = 2.49 × 10^− 9^, OR = 7.07, Fig. 4B and Additional file 2: Table S3). These results confirmed that the pseudotime analysis successfully reconstruct the process of osteoblast differentiation, and we therefore highlighted all genes significantly altered along pseudotime as key osteoblast lineage genes (Additional file 2: Table S4).

We hypothesized that these key lineage genes might be downstream targets of intercellular signaling networks, which regulated the process of osteoblast differentiation. Thus, we applied nichenetr network analysis [39] to prioritize the intercellular ligands and pathways that might be upstream of these key genes. Nichenetr prioritized 20 potential ligands that could regulate the expression of these key genes (Fig. 4C and Additional file 1: Figure S3), such as *IL1A* and *IL1B* that mainly secreted from macrophage, *TNF* and *APOE* that mainly secreted from monocyte, as well as *IFNG* and *TFGB1* that mainly secreted from T cell. *BMP2* and *BMP7*, top prioritized ligands that are known to regulate osteoblast activity [43], were mainly expressed in APSC. We then inferred the potential receptors on osteoblast that mediated these ligand-target associations (Fig. 4C), and found that *TGFBR1*, *FGFR3* and *TGFBR2* were linked with most key genes. Interestingly, ascending key genes were also enriched in GO term “response to fibroblast growth factor” (GO P = 1.82 × 10^− 7^, OR = 6.53, Fig. 4B). We managed all nichenetr-inferred regulation relations into a circus plot, showing the intact network underlying osteoblast maturation (Fig. 4C).

Taking one step further, we asked whether this regulation network involved any key transcription factors (TF). We applied SCENIC [40] to infer the TF-gene regulation network and found that eight key osteoblast lineage genes (such as *PTCH1*, *BMP7*, *EGR2*) functioned as TF that could regulate other key osteoblast lineage genes (Fig. 4D). They were downstream to receptor such as *LTBR*, *FRFR3*, *TGFBR2*, *BMPR1A* and *RARG*. By combination of ligand-receptor-TF-target regulation results, we identified several important pathways such as *FGF2-FGFR3-ID1-MMP2/MMP13/ALPL/TNC*, *TGFB1-TGFBR2-FOXQ1-HGF/FST/WNT5A*. Interestingly, we also observed that BMP could serve as both ligand and TF in the regulation of osteoblast differentiation, and regulated the expression of *PTCH1*, *LTBP1*, *EDNRA*. Taken together, our result generated a gene regulation network originated from cell type-specific ligands, which could regulate the osteoblast differentiation.

### *BMP-FGFR1-MSX1* pathway had central role in APSC renewal regulation

**Fig. 5:**
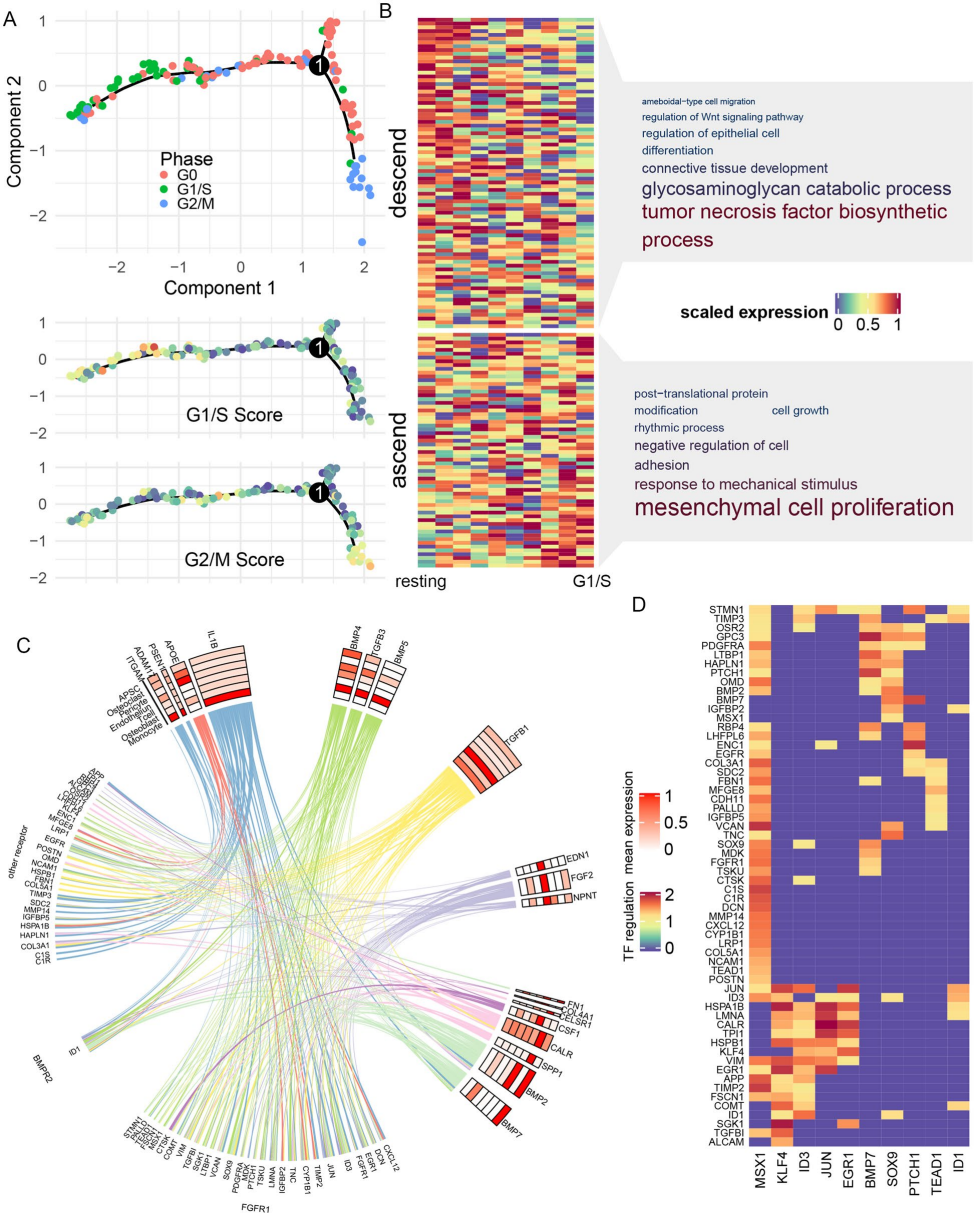
Intercellular signaling network regulating APSC self-renewal. **A** Pseudotime analysis of APSC. Lower panel showed the cell cycle score along pseudotime. **B** GO-BP analysis of gene significantly altered along pseudotime. Similar to Fig. 4B. **C** Circus plot of the signaling network, similar to Fig. 4C. **D** Regulation potentiality of TF on self-renewal related target genes

Apical papilla stem cell (APSC) resides in human tooth germ and retains multipotent and proliferation capacity via consistent self-renewal. To identify essential signaling pathways that regulate APSC renewal, we first applied monocle2 [44] pseudotime analysis to resolve the cell cycle alteration of APSC. As shown in Fig. 5A, APSC exhibited gradual transmission along cell cycle: on the right branch, APSC within G2/M phase gradually left cell cycle and transmitted into resting state, whereas in the left branch, G0 resting APSC transmitted into G1/S phase and entered cell cycle again. We took the left branch as the APSC renewal process and applied differential expression analysis to identified genes that altered along this process, such as *SERPING1*, *DKK3* and *HSPA1B* (Additional file 1: Figure S4). As shown in Fig. 5B and Additional file 2: Table S5, S6, genes that were up-regulated during renewal took part in mesenchymal cell proliferation (GO P = 3.67 × 10^−4^, OR = 30.3), cell growth (GO P = 8.82 × 10^−5^, OR = 6.89). On the other hand, genes related to tumor necrosis factor bio-synthesis (GO P = 9.98 × 10^−3^, OR = 21.4) and glycosaminoglycan catabolic (GO P = 8.77 × 10^−4^, OR = 19.9) were down regulated during APSC renewal. We took all differential expression genes together and defined them as APSC renewal genes (Additional file 2: Table S7).

We next applied Nichenetr to identify upstream intercellular signals that regulated the APSC renewal genes. As shown in Fig. 5C and Additional file 1: Figure S4, ligands released from monocytes (*IL1B* and *IL1A*), Osteoblast (*BMP4*, *TGFB3*, *BMP5*) and T cells (*TGFB1*) had the strongest regulation potentiality on APSC renewal genes. The autocrine of *BMP2* and *BMP7* on APSC also regulated a large number of renewal genes. These ligands mostly acted on APSC receptor *FGFR1*, which regulated 28 downstream renewal genes (Fig. 5C and Additional file 1: Figure S4), including *MSX1*, *PTCH1* and *SOX9*. Similar to the analysis of Osteoblast, we applied SCENIC analysis to highlight key TF in this ligand-target network (Fig. 5D). We found a transcription factor *MSX1*, which was a downstream target of *FGFR1*, regulated 47 renewal genes (Fig. 5D), especially *VCAN*, *C1S* and *TIMP2* (regulation score > 1.5). By taking all *MSX1* targets as a whole (so-called “regulon” [40]), we found that they were generally elevated during APSC transition to G1/S phase. These results highlighted the role of *BMP-FGFR1-MSX1* pathway in APSC renewal. In addition, we also found other TF such as *KLF4*, *ID3*, *JUN* and *EGR1*, that regulated other renewal genes like H*SPA1B*, *CALR*, *SGK1*.

### Transformation of osteoclast is regulated by signals from osteoblast and macrophage

**Fig. 6:**
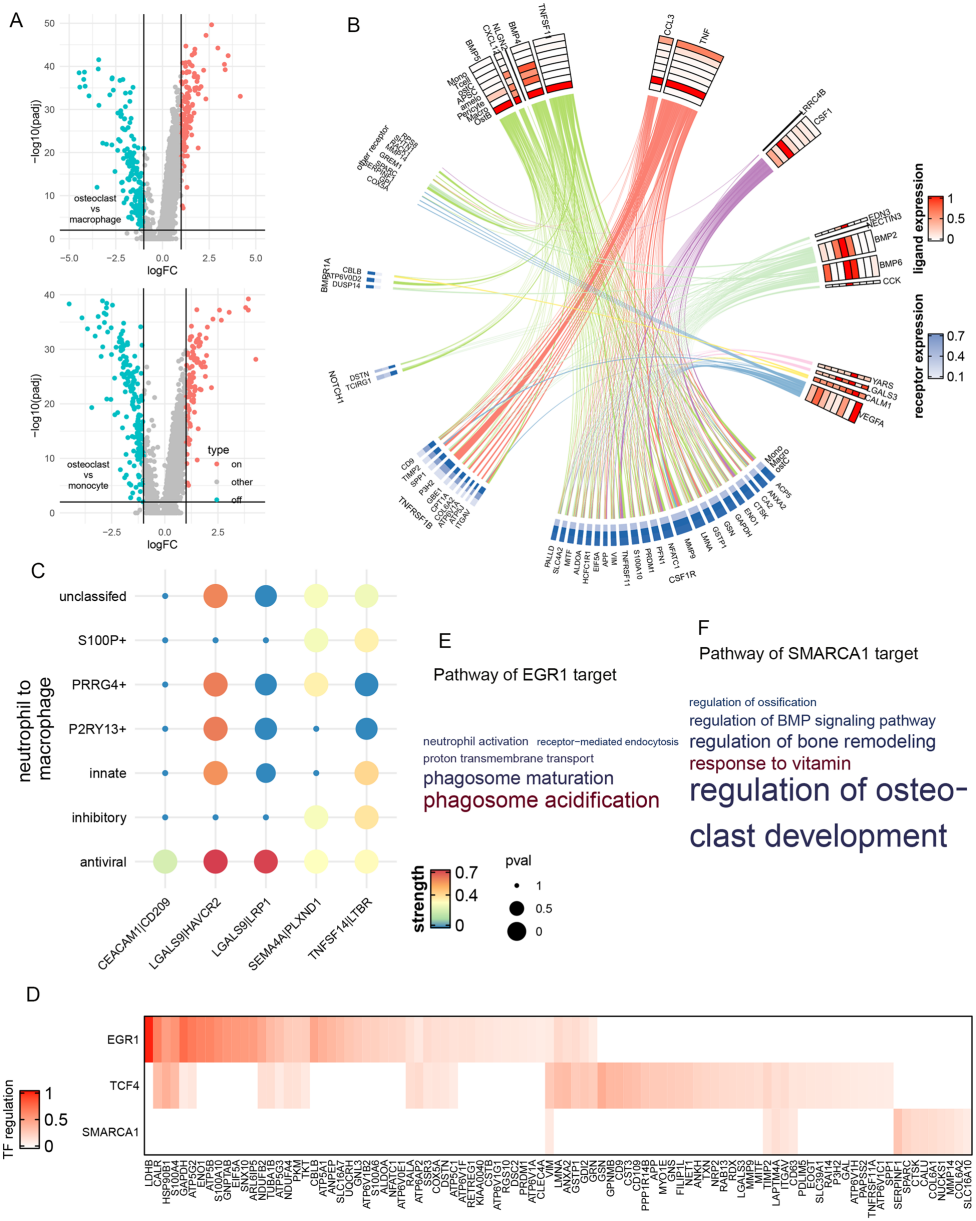
Intercellular signaling network regulating Osteoclast. **A** Differential expression analysis between osteoclast, macrophage and monocyte. **B** Circus plot of the signaling network, similar to Fig. 4 C. **C** CellphoneDB analysis of neutrophil-to-macrophage signaling pathway, similar to Fig. 2F. **D** Regulation potentiality of LTBR downstream TF on osteoclast transformation related target genes. **E**, **F** GO-BP analysis of target genes of EGR1 and SMARCA1

In tooth and other bone tissues, monocytes and macrophage continuously transform into osteoclasts, the dysregulation of which might disrupt the bone remodeling balance. To find the signaling pathways that regulate this transformation, we first identified transformation-related genes by differential expression analysis (Fig. 6A). At the significance threshold of false discovery rate (FDR)-adjusted P < 0.01 and log fold change > 1, we found 111 genes that were elevated during monocyte-to-osteoblast transformation and 149 genes that were elevated during macrophage-to-osteoblast transformation (Additional file 2: Tables S8, S9). We merged these two gene lists into 183 unique transformation-related genes and applied nichenetr [39] to discover the upstream signaling pathways that regulated them (Fig. 6B). In accordance with previous studies, ligands from osteoblasts (*BMP4*, *BMP5*, *TNFSF11*) and macrophage (*CCL3* and *TNF*) regulated the largest number of transformation-related genes (40 and 35, respectively, Additional file 1: Figure S5). *CSF1* secreted from pericyte also regulated 23 transformation-related genes via receptor *CSF1R*. Interestingly, the cellular distribution of receptors of these ligands were different: *BPMR1A* and *CSF1R* mainly expressed on macrophage and osteoclast, whereas *TNFRSF1B* and *NOTCH1* mainly expressed on monocytes (Fig. 6B). This result indicated that monocyte-to-osteoblast and macrophage-to-osteoblast transformation was regulated by different signaling pathways. Nonetheless, many key genes involved in osteolysis, such as *ACP5*, *NFATC1*, *MMP9*, were regulated by multiple signaling pathways (Additional file 1: Figure S5).

It has been revealed that neutrophil could activate osteoclasts and trigger osteonecrosis during inflammation. Since CellPhoneDB [32] analysis (Fig. 3D) found that neutrophil subtypes had dense connection with macrophage but few interactions with monocyte, we studied the neutrophil-to-macrophage signaling that could regulate the transformation-related genes. As shown in Fig. 6C, we found five ligand-receptor pathways that were significantly activated between neutrophil and macrophage. Among them, *TNFSF14-LTBR* pathway was shared by all seven neutrophil subtypes, whereas *CEACAM1-CD209* was specific to *MX1*^+^ antiviral neutrophils. We further explored the downstream TF regulation networks of these pathways by nichenetr and SCENIC, and found three TF whose target genes significantly enriched in transformation-related genes: *EGR1* (Fisher P = 8.90 × 10^−16^, OR = 5.32), *TCF4* (Fisher P = 5.94 × 10^− 15^, OR = 4.70) and *SMARCA1* (Fisher P = 0.02, OR = 2.11, Fig. 6D). Interestingly, they were all downstream to *TNFSF14-LTBR* signaling pathway, supporting its essential role in osteoclast transformation. These TFs regulated multiple osteoclast maturation markers [45], such as *CALR*, *MMP9*, *NFATC1*. We further applied GO analysis and found that the target genes of *EGR1* significantly enriched in functions related to osteolysis, such as phagosome acidification (GO P = 3.11 × 10^−5^, OR = 19.7) and receptor-mediated endocytosis (GO P = 3.26 × 10^−4^, OR = 4.10). For *SMARCA1*, the most significant enrichment was found for regulation of osteoclast development (GO P = 0.01, OR = 39.5), which suggested that *SMARCA1* may be one of the regulators of osteoclast transformation. For *TCF4* targets, we did not observe significant functional enrichment.

## Discussion

In the current study, we characterized the single-cell transcriptome of human tooth germ from growing third molar to decipher the cell subtype-specific signaling pathways that regulate the biological process of tooth development. We deciphered the subtypes of resident immune cells, refined the network of known tooth development regulators like *BMP*, *FGF* and *MSX1*, and discovered novel signaling pathways like.

The role of BMP family in the development and regulation of dental cells has long been highlighted by researchers. BMP family encodes various bone morphogenetic proteins, which consist of large subdivision of transforming growth factor-β ligand family [9]. In skeletal tissue, BMP regulates the osteoblastogenesis and extra-cellular matrix formation, whereas in dental tissue, BMP also regulate functions of dental pulp cells [10] and osteoclasts [14]. Following these observations, our hypothesis-free signaling network analysis further discovered that downstream pathways of these regulation were distinct. For the maturation of osteoblast, BMP regulated the osteoblast expression of *ID1* and *VCAN* via *BMP2-BMPR1A* signaling, in line with their known activities during osteoblastogenesis [9]. In the self-renewal of APSC, *BMP4* and *BMP5* secreted from osteoblast activated *FGFR1* and downstream *MSX1* to regulate a large number of renewal-related genes like *SOX9* and *ID3*. Concordantly, knock-down study on mice have demonstrated that *BMP4* and *MSX1* are essential in tooth organogenesis [11]. In the transformation of osteoclast from monocyte and macrophage, the role of BMP is less significant than major regulators *CSF1*, *TNF* and *TNFRSF11*, but the *BMP4/2-BMPR2* pathway still showed regulation potentiality on osteoclast genes like *SPP1* and *GREM1*. This result suggested that while BMP family took part in various biological process of tooth, the mechanism of each process is distinct and should be analyzed separately. Concordantly, each member of BMP family also showed distinct roles in different process of dental development, and their functions might be from multi-aspect. For example, *BMP7* secreted from APSC could serve as ligand to act on osteoblast, whereas *BMP7* expressed in osteoblast could also serve as transcription factors and regulate osteoblast maturation.

We also identified the complex regulation network initiated by *TGF* and *FGF*, in accordance with their known roles in tooth development. Aside from BMP family, the transforming growth factor-β (TGFB) ligand family includes various genes including *TGFB1*, *TGFB2* and *TGFB3 *[13]. In dental tissue, TGFB ligands regulate the pulpal repair and dentinogenesis, possibly through the *SMAD2* and extracellular signal-regulated kinases (ERK) pathways in pulp cell [12]. In our single-cell analysis, we further found that *TGFB1*, which was mainly originated from T cells, activated receptor *TGFBR1* and *TGFBR2* to regulate a large number of genes involved in osteoblast maturation. This regulation was in part mediated by transcription factor *FOXQ1*. As for APSC renewal, both *TGFB1* and *TGFB3* (mainly originated from osteoblast) showed high regulation potentiality. Similarly, we also found the cell-type specific networks of FGF signaling, which was known to play a role in tooth development but the intact pathway remained to be elucidated [46]. Specifically, receptor *FGFR3* was involved in osteoblast maturation, whereas *FGFR1* was mainly responsible for ASPC self-renewal. The downstream transcription factor (*ID1* and *MSX1*, respectively) was also distinct in these two processes. Another interesting fact is that aside from *FGF2*, ligands *BMP2*, 4 and 5 also showed affinity with FGF receptor and exhibited even higher regulation potentiality, highlighting the importance of cross-pathway signaling transduction.

Our analysis also discovered the role of immune cell in dental development, which was not strengthened by previous study. For example, *IL1B*, which was mainly expressed in monocyte, regulated 34 APSC renewal-related genes, more than other non-immune ligands could regulate. It also regulated the maturation of osteoblast together with *IL1A*. It is known that lymphocytes could inhibit the dental pulp development by secreting cytokines like *IL1B* and *IL6*, but this inhibition was only found in inflammatory status [47]. Alternatively, since the tooth germ sample in the current study was collected at normal status, our result suggested that *IL1B* could also regulate the normal developmental process of human tooth in the absence of inflammation. Similarly, *TGFB1* also regulated 39 renewal-related genes, and was mainly expressed in T cells. As for osteoclast transformation, the top ligand was *TNF* from macrophage. These results indicated that dental immune cells not only defense against pathogens, but also regulate the dental development via secretion of ligands that act on other dental cell types. Under the situation of inflammation or stress, such regulation might lean towards suppression of osteogenesis and activation of osteoporosis.

It should be noted that since dental germ developmental stages exhibit high heterogeneity, and that samples collected from different individual may represent distinct status and could not cover the entire process of dental development. As a consequence, our result mainly complemented the up- and down-stream components of known signaling networks like BMP and FGF pathway, but did not reveal novel and unknown pathways. For example, the samples analyzed in the current study contained relatively small number of ameloblasts and odontoblasts, which are mainly activated at the early stage of dental development. Since it is relatively difficult to obtain highly immature dental tissue in clinical scenario, the signaling networks of these cell types might be better analyzed using prenatal tissues or by translational studies of mouse model. Compared with the current study, two previous single-cell analysis [16, 48] that aimed at different anatomical structures of adult tooth have revealed different cell components and provided insights into different biological process of mature tooth. In the future, more efforts should be devoted to the single-cell analysis of human embryo dental tissues.

In conclusion, we provided a cell interactome landscape for postnatal human germ and discovered the key signaling pathways regulating the development of dental cells, which provided novel insights into the mechanism of dental development and highlighted potential targets for disease intervention and dental regeneration.

## Method

### Sample collection and pre-processing

This study was approved and supervised by Ethical committee of Shanghai Ninth People’s Hospital. Written informed consent was provided by all participants. We ruled out the possibility of infection based on the following observation: the third molars of the two patients had no history of pain or infection, and the molar did not erupt, the surrounding gums and alveolar mucosa were healthy, without redness, swelling, and fistula, or other signs of infection. On oral panorama, there was no abnormal low-density shadow around the third molars of the two patients. Blood examination also revealed no sign of infection. Both the surgical procedures were performed by the same surgeon and assistant with patients under local anesthesia. After a full-thickness mucoperiosteal flap elevation, the buccal bone of the third molar was removed with piezosurgery handpiece to expose the tooth germ, which was carefully enucleation by curette immediately. We performed the crown sectioning with a high-speed handpiece and fissure burs to remove the calcified structures when necessary. The tooth germ free of mineralized part was rinsed with normal saline and stored in MACS Tissue Storage Solution (Miltenyi, German), and was immediately delivered to Single-cell RNA-seq platform within 10 min.

### Single-cell dissociation

Single-cell RNA-seq experiment was performed by experimental personnel in the laboratory of NovelBio Bio-Pharm Technology Co Ltd. Briefly, samples were first washed with phosphate-buffered saline (PBS), minced into small pieces (approximately 1 mm^3^) on ice and enzymatically digested with 0.5 mg/mL collagenase I/II (Worthington) and 50 U/mL DNase I (Worthington) for 45 min at 37 °C, with agitation. After digestion, samples were sieved through a 70 μm cell strainer, and centrifuged at 4 °C, 300×*g*, 5 min, to remove the supernatant. The pelleted cells were suspended in red blood cell lysis buffer (Miltenyi Biotec) to lyse red blood cells. After washing with PBS containing 0.04% BSA, the cell pellets were re-suspended in PBS containing 0.04% BSA and re-filtered through a 35 μm cell strainer. Dissociated single cells were then stained for viability assessment using Calcein-AM (Thermo Fisher Scientific) and Draq7 (BD Biosciences). The single-cell suspension was further enriched with a MACS dead cell removal kit (Miltenyi Biotec) [49].

### Single-cell RNA sequencing

BD Rhapsody system was used to capture the transcriptomic information of the (two sample-derived) single cells. Single-cell capture was achieved by random distribution of a single-cell suspension across > 200,000 microwells through a limited dilution approach. Beads with oligonucleotide barcodes were added to saturation so that a bead was paired with a cell in a microwell. The cells were lysed in the microwell to hybridize mRNA molecules to barcoded capture oligos on the beads. Beads were collected into a single tube for reverse transcription and ExoI digestion. Upon cDNA synthesis, each cDNA molecule was tagged on the 5′ end (that is, the 3′ end of a mRNA transcript) with a unique molecular identifier (UMI) and cell barcode indicating its cell of origin. Whole transcriptome libraries were prepared using the BD Rhapsody single-cell whole-transcriptome amplification (WTA) workflow including random priming and extension (RPE), RPE amplification PCR and WTA index PCR. The libraries were quantified using a High Sensitivity DNA chip (Agilent) on a Bioanalyzer 2200 and the Qubit High Sensitivity DNA assay (Thermo Fisher Scientific). Sequencing was performed by illumina sequencer (Illumina, San Diego, CA) on a 150 bp paired-end run.

### Single-cell RNA analysis

scRNA-seq-seq data analysis was performed by NovelBio Bio-Pharm Technology Co., Ltd. with NovelBrain Cloud Analysis Platform. We applied fastp [50] with default parameter filtering the adaptor sequence and removed the low-quality reads to achieve the clean data. UMI-tools [51] were applied for Single Cell Transcriptome Analysis to identify the cell barcode whitelist. The UMI-based clean data was mapped to human genome (Ensemble version 91) utilizing STAR v2.7.6 [52] mapping with customized parameter from UMI-tools standard pipeline to obtain the UMIs counts of each sample. Cells contained over 200 expressed genes and mitochondria UMI rate below 20% passed the cell quality filtering and mitochondria genes were removed in the expression table. Seurat v3.0 package [21] was used for cell normalization and regression based on the expression table according to the UMI counts of each sample and percent of mitochondria rate to obtain the scaled data. PCA was constructed based on the scaled data with top 2000 high variable genes and top 10 principals were used for tSNE construction and UMAP construction. We calculated cell cycle score using cell cycle gene lists from Tirosh et al. [53] and included this score in the normalization.

We applied graph-based Louvain cluster method with resolution = 0.8, we acquired the unsupervised cell cluster result based the PCA top 10 principal and we calculated the marker genes by FindAllMarkers function with wilcox rank sum test algorithm under default criteria. We also applied the same function to find genes significantly differed between osteoclast, macrophage and monocyte. For T cell subtypes, we reran FindVariableGenes and PCA, reran clustering analysis with top 13 PCA and resolution = 0.6. For SOX9^+^ cells, osteoblasts and Neutrophils, we also carried out similar second-level clustering analysis.

### CellPhoneDB analysis

we applied cell communication analysis based on the CellPhoneDB [32], a public repository of ligands, receptors and their interactions. Membrane, secreted and peripheral proteins of the cluster of different time point was annotated. Significant mean and Cell Communication significance was calculated based on the interaction and the normalized cell matrix with permutation test. For each cell pair, we gathered all ligand-receptor pairs with nominal p < 0.05.

### Pseudotime analysis

We applied the Single-Cell Trajectories analysis utilizing Monocle3 [41] for osteoblast pseudotime analysis. The pre-processing, dimension reduction, clustering and trajectory reconstruction were run with default parameter, using all available genes. A specific branch occurred at the midpoint of trajectory in Fig. 4A, but differential expression analysis did not reveal specific biological characteristics of it. We reasoned that this branch might reflect osteoblasts without common expression pattern of maturation-related genes, possibly due to drop-out, and did not further analyze them. For APSC, we used differential GeneTest to find high variation genes that highly correlated with cell cycle scores and applied trajectory analysis on them using Monocle2 [44] DDR-Tree and default parameter. We did not apply Monocle3 to APSC since the learn_graph() function does not support pseudotime analysis using only a subset of genes, as we applied with the cell cycle analysis.

After we calculated the pseudotime for each cell, we applied differential GeneTest function to find genes that significantly altered along pseudotime. According to the trends of alteration (i.e., sign of Pearson Correlation Coefficient between gene expression and pseudotime), we separated these genes into ascending and descending genes. We then applied Gene Ontology Biological Process (GO-BP) analysis by clusterProfiler [54] R package separately on these genes to elucidate their functions.

### NicheNet analysis

Having identified the key gene lists in osteoblast maturation, APSC self-renewal and osteoclast transformation, we applied NicheNet [39] analysis to find the regulation network upstream of these gene sets. NicheNet has constructed a priori networks consisting of ligands, receptors and targets. Given a set of targets and the range of expressed ligands and receptors, NicheNet finds the ligands and receptors showing the highest regulation potentiality on them. In the current study, NicheNet was applied with the following parameter: threshold of expression = 25% in receptor cell and 10% in sender cell, number of ligand-target pairs = 100, ligand-target activity threshold = 0.33. For uniformity, we reported top 20 ligands for all analysis.

For each of the highlighted ligand and receptor, we calculated their average expression in the corresponding cell clusters. We denoted the origin of each ligand as the cell cluster showing the highest average expression. For the ease of visualization, we aligned each target to only one receptor with the highest regulation potentiality in the a priori network. All targets that were predicted to be regulated by at least one of the ligands, but did not had potential upstream receptors, were labelled “other receptor” in the circus plot.

### SCENIC analysis

To assess transcription factor regulation strength, we applied the Single-cell regulatory network inference and clustering (pySCENIC, v0.9.5) [40] workflow, using the 20-thousand motifs database for RcisTarget and GRNboost. A regulation score > 1 calculated by SCENIC was taken as evidence of the regulation activity of the corresponding TF and target genes. To select TF of interest, we only included those TF downstream of the NicheNet ligands or receptors, as denoted by the a priori network of NicheNet.

### Statistical analysis

Statistical analysis was carried out in R 4.0 (R Core Team). All p values were two-tailed unless otherwise specified. For the comparison of gene expression between cell clusters, we applied permutation test by coin R package [55]. For the enrichment of TF targets in specific gene lists, we applied Fisher exact test with background gene list defined as all genes with regulation score > 1.

## Supplementary Information

**Additional file 1: Figure S1.** Full oral panorama for stage A (up) and stage D (down) toothgerm, corresponding to Fig. 1A. **FigureS2.** H-E staining of tooth germ slices, corresponding to Fig. 1F (up) andFig. 1G (down). Upper panel indicated osteoblast morphology, and lower panelindicated odontoblast morphology. **FigureS3.** **A** Pseudotime trajectory forselected gene expression. **B** Ligand-receptoractivity predicted by nichenetr. **C** Ligand-targetactivity predicted by nichenet. **FigureS4.** Similar to Figure S3, but for APSC self-renewal. **Figure S5.** Similar to Figure S3, but for osteoclast transformation.

**Additional file 2.** **Table S1 to S9.**

## Data Availability

Expression data will be made available at https://github.com/WeiCSong upon publication.

## References

[1] Balic A (2019). Concise review: cellular and molecular mechanisms regulation of tooth initiation. Stem Cells.

[2] Balic A, Thesleff I (2015). Tissue interactions regulating tooth development and renewal. Current topics in developmental biology.

[3] Thesleff I (2014). Current understanding of the process of tooth formation: transfer from the laboratory to the clinic. Aust Dent J.

[4] Matalová E, Lungová V, Sharpe P (2015). Development of tooth and associated structures. Stem cell biology and tissue engineering in dental sciences.

[5] Seppala M, Fraser GJ, Birjandi AA, Xavier GM, Cobourne MT (2017). Sonic hedgehog signaling and development of the dentition. J Dev Biol.

[6] Liu F, Millar SE (2010). Wnt/β-catenin signaling in oral tissue development and disease. J Dent Res.

[7] Du W, Du W, Yu H (2018). The role of fibroblast growth factors in tooth development and incisor renewal. Stem Cells Int.

[8] Niwa T, Yamakoshi Y, Yamazaki H, Karakida T, Chiba R, Hu JCC (2018). The dynamics of TGF-β in dental pulp, odontoblasts and dentin. Sci Rep.

[9] Lowery JW, Rosen V (2018). The BMP pathway and its inhibitors in the skeleton. Physiol Rev.

[10] Chakka LRJ, Vislisel J, Vidal CDMP, Biz MT, Salem K, Cavalcanti A (2020). Application of BMP-2/FGF-2 gene-activated scaffolds for dental pulp capping. Clin Oral Investig.

[11] Jia S, Kwon HJE, Lan Y, Zhou J, Liu H, Jiang R (2016). Bmp4-Msx1 signaling and Osr2 control tooth organogenesis through antagonistic regulation of secreted Wnt antagonists. Dev Biol.

[12] Chang MC, Chang HH, Lin PS, Huang YA, Chan CP, Tsai YL (2018). Effects of TGF-β1 on plasminogen activation in human dental pulp cells: role of ALK5/Smad2, TAK1 and MEK/ERK signalling. J Tissue Eng Regen Med.

[13] Morikawa M, Derynck R, Miyazono K (2016). TGF- β and the TGF-β family: context-dependent roles in cell and tissue physiology. Cold Spring Harbor Perspect Biol.

[14] Omi M, Kaartinen V, Mishina Y (2019). Activin A receptor type 1-mediated BMP signaling regulates RANKL-induced osteoclastogenesis via canonical SMADsignaling pathway. J Biol Chem.

[15] Yu S, Li J, Zhao Y, Li X, Ge L (2020). Comparative secretome analysis of mesenchymal stem cells from dental apical papilla and bone marrow during early odonto/osteogenic differentiation: potential role of transforming growth factor-β2. Front Physiol.

[16] Chiba Y, Saito K, Martin D, Boger ET, Rhodes C, Yoshizaki K (2020). Single-cell RNA-sequencing from mouse incisor reveals dental epithelial cell-type specific genes. Front Cell Dev Biol.

[17] Krivanek J, Soldatov RA, Kastriti ME, Chontorotzea T, Herdina AN, Petersen J (2020). Dental cell type atlas reveals stem and differentiated cell types in mouse and human teeth. Nat Commun.

[18] Takahashi A, Nagata M, Gupta A, Matsushita Y, Yamaguchi T, Mizuhashi K (2019). Autocrine regulation of mesenchymal progenitor cell fates orchestrates tooth eruption. Proc Natl Acad Sci USA.

[19] Qian S, Huang Q, Shi J, Zhou L, Zhao Y, Li B (2021). Single-cell RNA sequencing identifies heterogeneous cell subtypes within gingival tissue. Res Sq.

[20] Demirjian A, Goldstein H, Tanner JM (1973). A new system of dental age assessment. Hum Biol.

[21] Stuart T, Butler A, Hoffman P, Hafemeister C, Papalexi E, Mauck WM (2019). Comprehensive integration of single-cell data. Cell.

[22] Rosset EM, Bradshaw AD (2016). SPARC/osteonectin in mineralized tissue. Matrix Biol.

[23] Li D, Cai L, Meng R, Feng Z, Xu Q (2020). METTL3 modulates osteoclast differentiation and function by controlling RNA stability and nuclear export. Int J Mol Sci.

[24] Aguilar P, Lertchirakarn V (2016). Comparison of stem cell behaviors between indigenous high and low-CD24% expressing cells of stem cells from apical papilla (SCAPs). Tissue Cell.

[25] French LE, Chonn A, Ducrest D, Baumann B, Belin D, Wohlwend A (1993). Murine clusterin: molecular cloning and mRNA localization of a gene associated with epithelial differentiation processes during embryogenesis. J Cell Biol.

[26] Lian N, Wang W, Li L, Elefteriou F, Yang X (2009). Vimentin inhibits ATF4-mediated Osteocalcin transcription and osteoblast differentiation. J Biol Chem.

[27] Chaible LM, Sanches DS, Cogliati B, Mennecier G, Dagli MLZ (2011). Delayed osteoblastic differentiation and bone development in Cx43 knockout mice. Toxicol Pathol.

[28] Tsukasaki M, Komatsu N, Nagashima K, Nitta T, Pluemsakunthai W, Shukunami C (2018). Host defense against oral microbiota by bone-damaging T cells. Nat Commun.

[29] Kaech SM, Tan JT, Wherry EJ, Konieczny BT, Surh CD, Ahmed R (2003). Selective expression of the interleukin 7 receptor identifies effector CD8 T cells that give rise to long-lived memory cells. Nat Immunol.

[30] Lyu M, Li Y, Hao Y, Lyu C, Huang Y, Sun B (2019). CCR6 defines a subset of activated memory T cells of Th17 potential in immune thrombocytopenia. Clin Exp Immunol.

[31] Woodward Davis AS, Roozen HN, Dufort MJ, DeBerg HA, Delaney MA, Mair F (2019). The human tissue-resident CCR5+ T cell compartment maintains protective and functional properties during inflammation. Sci Transl Med.

[32] Efremova M, Vento-Tormo M, Teichmann SA, Vento-Tormo R (2020). CellPhoneDB: inferring cell–cell communication from combined expression of multi-subunit ligand–receptor complexes. Nat Protoc.

[33] Bozec A, Zaiss MM, Kagwiria R, Voll R, Rauh M, Chen Z (2014). T cell costimulation molecules CD80/86 inhibit osteoclast differentiation by inducing the IDO/tryptophan pathway. Sci Transl Med.

[34] Farges JC (2009). Understanding dental pulp innate immunity—a basis for identifying new targets for therapeutic agents that dampen inflammation. J Appl Oral Sci.

[35] Yang J, Masters S (2018). Human peptidoglycan recognition protein 1 in innate immunity. Cogent Biol.

[36] Pillai PS, Molony RD, Martinod K, Dong H, Pang IK, Tal MC (2016). Mx1 reveals innate pathways to antiviral resistance and lethal influenza disease. Science.

[37] Zabieglo K, Majewski P, Majchrzak-Gorecka M, Wlodarczyk A, Grygier B, Zegar A (2015). The inhibitory effect of secretory leukocyte protease inhibitor (SLPI) on formation of neutrophil extracellular traps. J Leukoc Biol.

[38] Girbl T, Lenn T, Perez L, Rolas L, Barkaway A, Thiriot A (2018). Distinct compartmentalization of the chemokines CXCL1 and CXCL2 and the atypical receptor ACKR1 determine discrete stages of neutrophil diapedesis. Immunity.

[39] Browaeys R, Saelens W, Saeys Y (2020). NicheNet: modeling intercellular communication by linking ligands to target genes. Nat Methods.

[40] Aibar S, González-Blas CB, Moerman T, Huynh-Thu VA, Imrichova H, Hulselmans G (2017). SCENIC: single-cell regulatory network inference and clustering. Nat Methods.

[41] Cao J, Spielmann M, Qiu X, Huang X, Ibrahim DM, Hill AJ (2019). The single-cell transcriptional landscape of mammalian organogenesis. Nature.

[42] Sterner RM, Kremer KN, Dudakovic A, Westendorf JJ, van Wijnen AJ, Hedin KE (2018). Tissue-nonspecific alkaline phosphatase is required for MC3T3 osteoblast-mediated protection of acute myeloid leukemia cells from apoptosis. J Immunol.

[43] Yamaguchi A, Sakamoto K, Minamizato T, Katsube K, Nakanishi S (2008). Regulation of osteoblast differentiation mediated by BMP, Notch, and CCN3/NOV. Jpn Dent Sci Rev.

[44] Qiu X, Mao Q, Tang Y, Wang L, Chawla R, Pliner HA (2017). Reversed graph embedding resolves complex single-cell trajectories. Nat Methods.

[45] Adapala NS, Barbe MF, Langdon WY, Nakamura MC, Tsygankov AY, Sanjay A (2010). The loss of Cbl-phosphatidylinositol 3-kinase interaction perturbs RANKL-mediated signaling, inhibiting bone resorption and promoting osteoclast survival. J Biol Chem.

[46] Li CY, Prochazka J, Goodwin AF, Klein OD (2014). Fibroblast growth factor signaling in mammalian tooth development. Odontology.

[47] Zhang J, Zhang Y, Lv H, Yu Q, Zhou Z, Zhu Q (2013). Human stem cells from the apical papilla response to bacterial lipopolysaccharide exposure and anti-inflammatory effects of nuclear factor IC. J Endod.

[48] Pagella P, de Vargas Roditi L, Stadlinger B, Moor AE, Mitsiadis TA (2021). A single-cell atlas of human teeth. iScience.

[49] Denisenko E, Guo BB, Jones M, Hou R, de Kock L, Lassmann T (2020). Systematic assessment of tissue dissociation and storage biases in single-cell and single-nucleus RNA-seq workflows. Genome Biol.

[50] Chen S, Zhou Y, Chen Y, Gu J (2018). Fastp: an ultra-fast all-in-one FASTQ preprocessor. Bioinformatics.

[51] Smith T, Heger A, Sudbery I (2017). UMI-tools: modeling sequencing errors in unique molecular identifiers to improve quantification accuracy. Genome Res.

[52] Dobin A, Davis CA, Schlesinger F, Drenkow J, Zaleski C, Jha S (2013). STAR: ultrafast universal RNA-seq aligner. Bioinformatics.

[53] Tirosh I, Izar B, Prakadan SM, Wadsworth MH, Treacy D, Trombetta JJ (2016). Dissecting the multicellular ecosystem of metastatic melanoma by single-cell RNA-sEq. Science.

[54] Yu G, Wang L-G, Han Y, He Q-Y (2012). clusterProfiler: an R package for comparing biological themes among gene clusters. Omics J Integr Biol.

[55] Hothorn T, Hornik K, van de Wiel MA, Zeileis A (2008). Implementing a class of permutation tests: the coin package. J Stat Softw.

