# Supplementary material for: A single-cell interactome of human tooth germ from growing third molar elucidates signaling networks regulating dental development
Source: Cell Biosci. 2021 Oct 2;11:178. doi: 10.1186/s13578-021-00691-5 (PMC8487529; doi:10.1186/s13578-021-00691-5)
Supplement: Supplementary file 1 — Additional file 1: Figure S1. Full oral panorama for stage A (up) and stage D (down) toothgerm, corresponding to Fig. 1A. FigureS2. H-E staining of tooth germ slices, corresponding to Fig. 1F (up) andFig. 1G (down). Upper panel indicated osteoblast morphology, and lower panelindicated odontoblast morphology. FigureS3. A Pseudotime trajectory forselected gene expression. B Ligand-receptoractivity predicted by nichenetr. C Ligand-targetactivity predicted by nichenet. FigureS4. Similar to Figure S3, but for APSC self-renewal. Figure S5. Similar to Figure S3, but for osteoclast transformation. [file 13578_2021_691_MOESM1_ESM.docx]

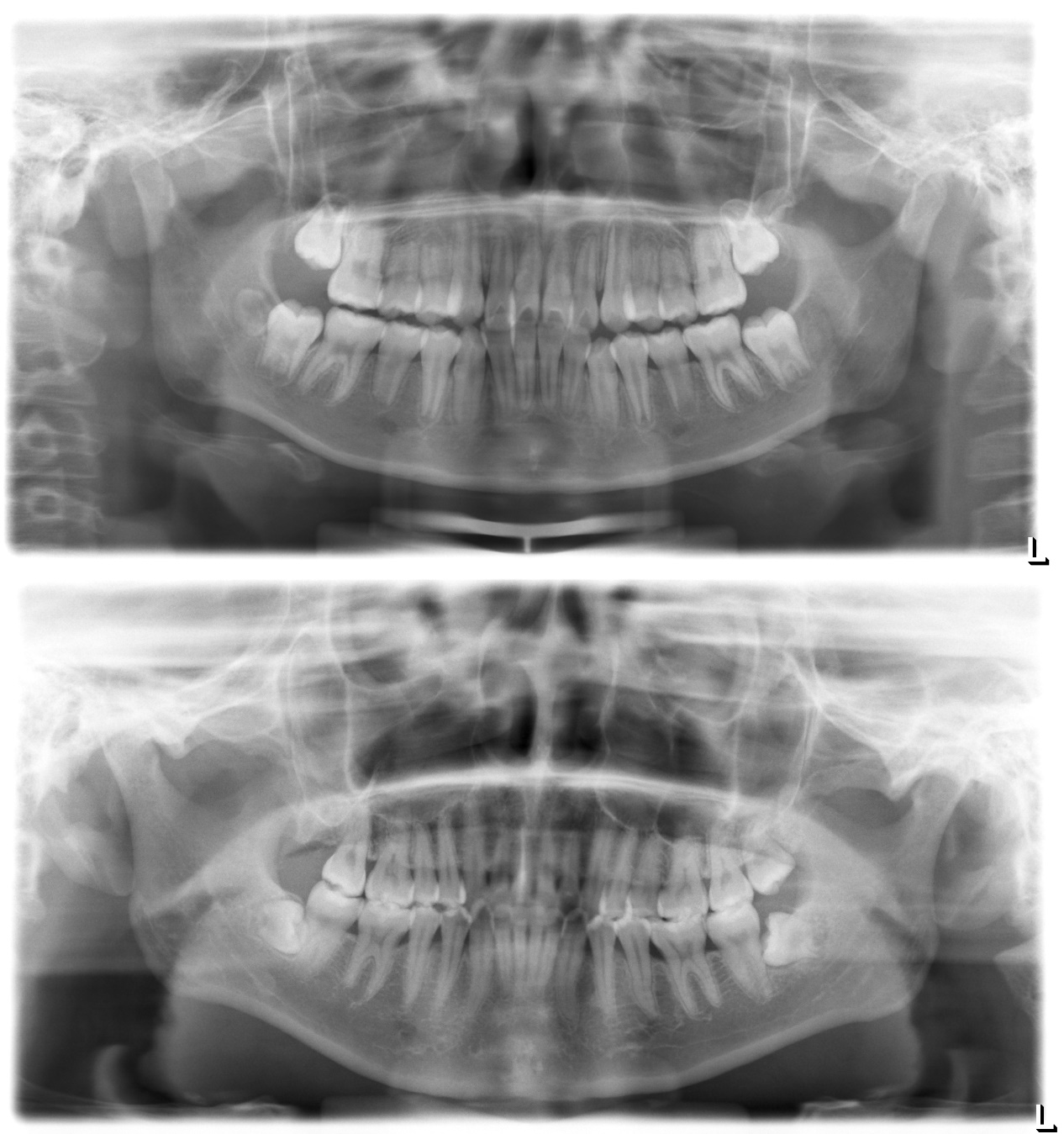


Figure S1. Full oral panorama for stage A (up) and stage D (down) tooth germ, corresponding to Figure 1A.


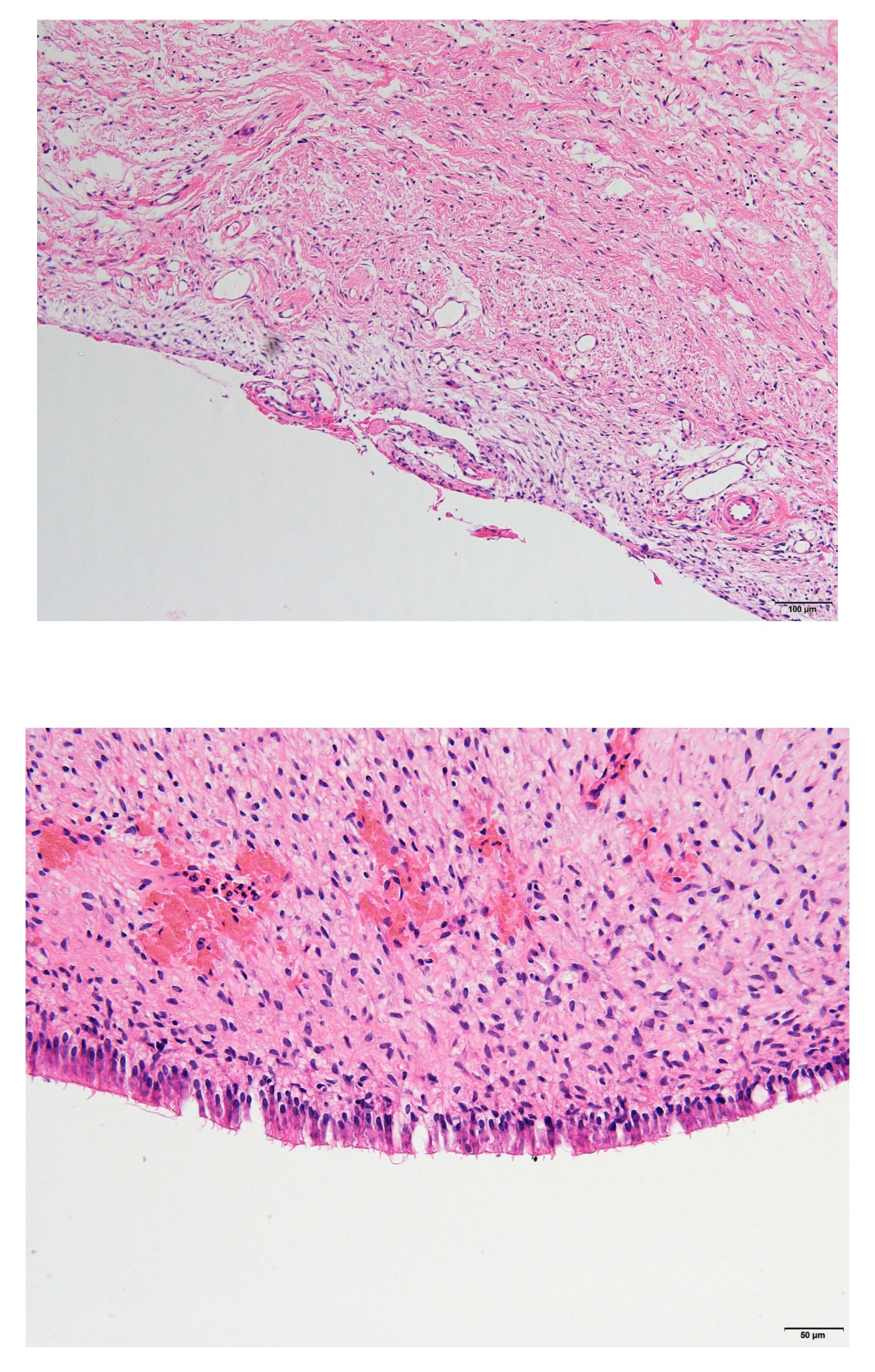


Figure S2. H-E staining of tooth germ slices, corresponding to Figure 1F (up) and Figure 1G (down). Upper panel indicated osteoblast morphology, and lower panel indicated odontoblast morphology.


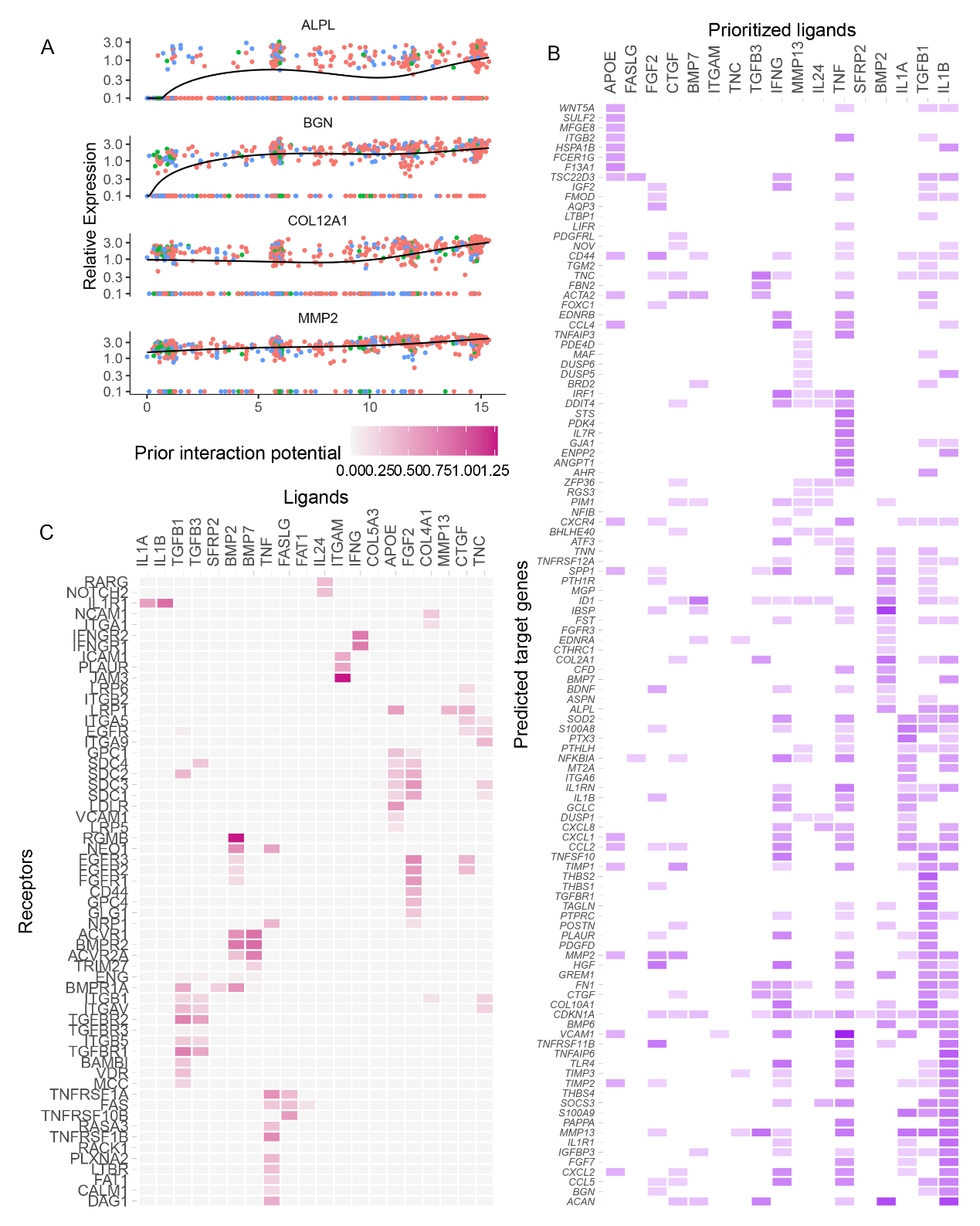


Figure S3. A: pseudotime trajectory for selected gene expression. B: ligand-receptor activity predicted by nichenetr. C: ligand-target activity predicted by nichenet.


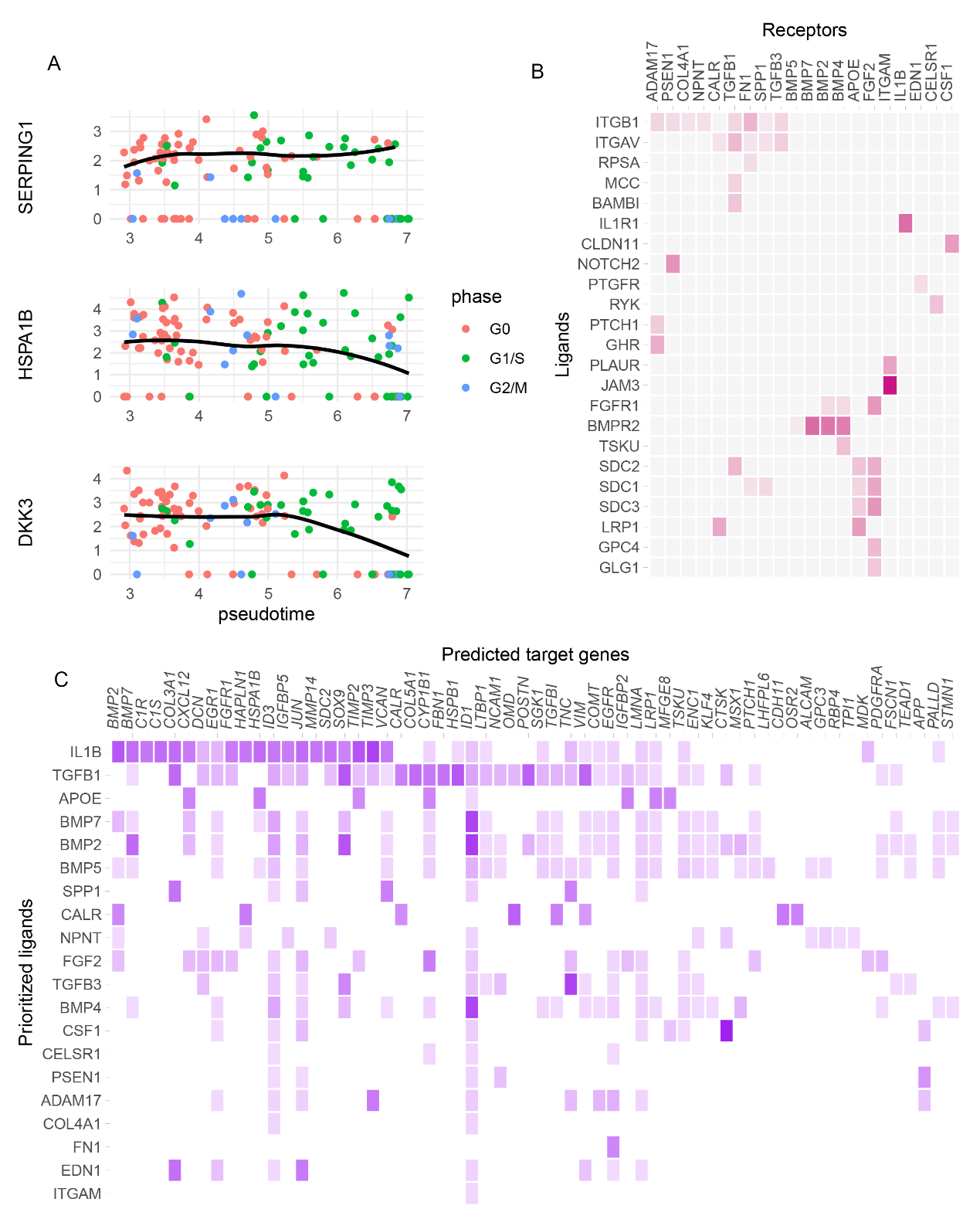


Figure S4. Similar to Figure S3, but for APSC self-renewal.


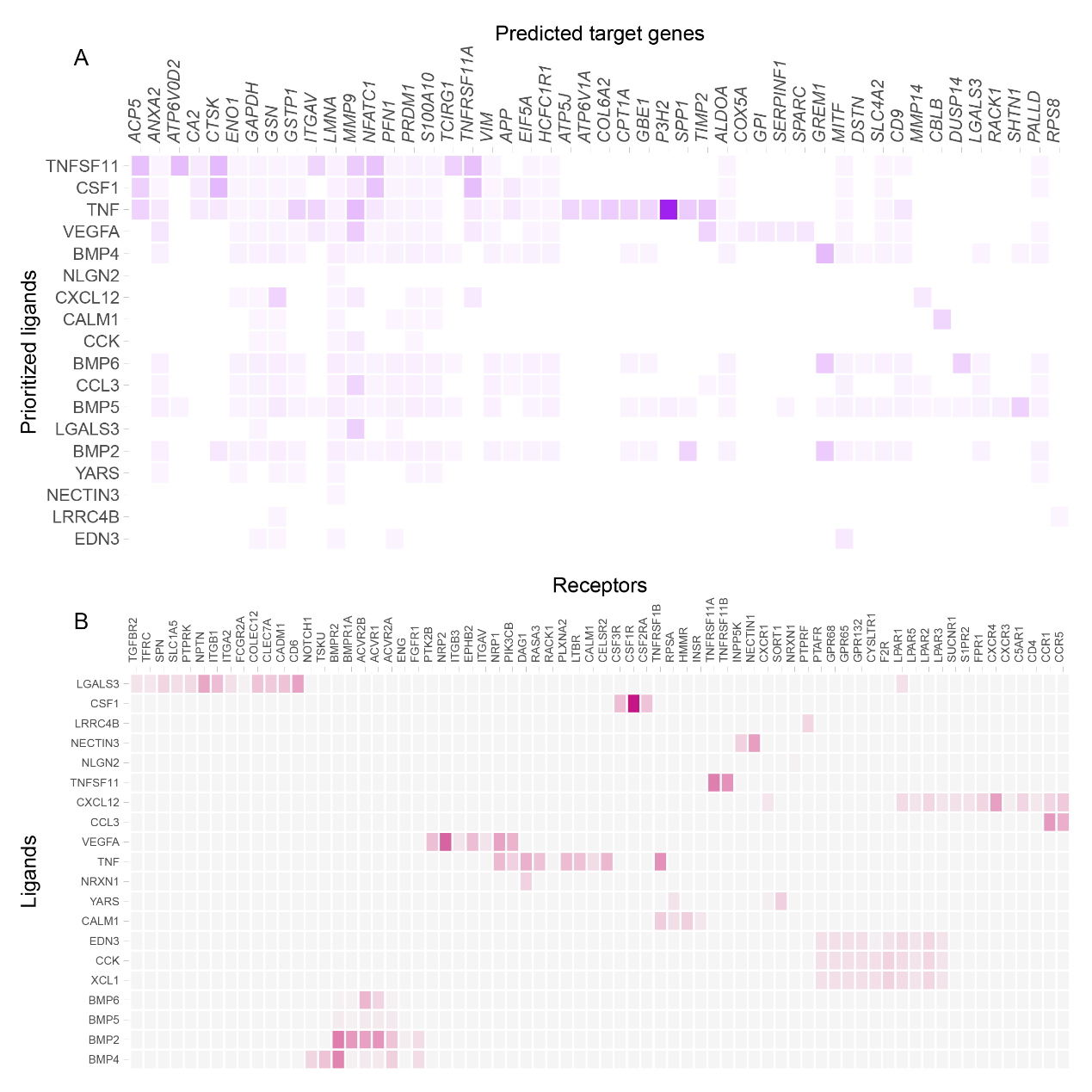


Figure S5. Similar to Figure S3, but for osteoclast transformation.
